# Inhibitory Effect and Mechanism of Hexanal on the Maturation of Peach-Shaped *Phallus impudicus*

**DOI:** 10.3390/jof11020127

**Published:** 2025-02-08

**Authors:** Hong He, Shuya Fan, Gan Hu, Beibei Wang, Dayu Liu, Xinhui Wang, Jinqiu Wang, Fang Geng

**Affiliations:** 1 School of Food and Biological Engineering, Chengdu University, Chengdu 610106, China; hehong@cdu.edu.cn (H.H.); shuya_fan@163.com (S.F.); liudayu@cdu.edu.cn (D.L.); wangxinhui@cdu.edu.cn (X.W.); 2 Institute for Advanced Study, Chengdu University, Chengdu 610106, China; hugan@cdu.edu.cn (G.H.); wangbeibei@cdu.edu.cn (B.W.); 3 National Agricultural Science and Technology Center, Chengdu 610213, China

**Keywords:** peach-shaped *phallus impudicus*, hexanal treatment, inhibition of maturation, quantitative transcriptome, lipidomic analysis

## Abstract

*Phallus impudicus* is a fungus used as a medicine and nutrient-rich food. However, the shelf life of mature *Phallus impudicus* is only a few hours. Therefore, research on its preservation technology is essential for improving its economic value. This study investigated the effects of hexanal concentrations (25–100 μL/L) and treatment time (4–8 h) on the inhibition of peach-shaped *Phallus impudicus* (CK) maturation and found that the maturation rate was 25% under optimal conditions of 25 μL/L hexanal treatment for 6 h. Quantitative transcriptomic and lipidomic analyses were conducted among CK, mature *Phallus impudicus* (M-P), and hexanal-treated peach-shaped *Phallus impudicus* (H-P-P). In total, 2933 and 2746 differentially expressed genes (DEGs) and 156 and 111 differentially abundant lipids (DALs) were identified in CK vs. H-P-P and M-P vs. H-P-P, respectively. Functional analysis demonstrated that hexanal treatment inhibited phospholipase D gene expression and reduced phosphatidic acid abundance, thereby inhibiting the activation of the phosphatidylinositol signaling system and the signal amplification of the cell wall integrity mitogen-activated protein kinase pathway. These blocked signal transductions inhibited the gene expression of most β-glucanases, chitinases and chitin synthases, further affecting cell wall reconstruction. Moreover, hexanal treatment enhanced membrane stability by reducing the monogalactosyl diglyceride/digalactosyl diacylglycerol ratio and increasing the phosphatidylcholine/phosphatidylethanolamine ratio. This study contributed to the development of hexanal treatment as a postharvest preservation technology for *Phallus impudicus*.

## 1. Introduction

*Phallus impudicus* (*P. impudicus*), a crypto fungus, contains multiple bioactive components, such as polysaccharides and proteins, and is called queen of mushrooms because of its unique taste, rich nutrition, and high medicinal value [1]. *P. impudicus* is widely consumed as an edible and medicinal fungus in Asian countries, including China, India, Japan, and so on, and has a long cultivation history in China, such as Yunnan, Sichuan, Guizhou, etc., [1,2]. There are four major growth phases of *P. impudicus*, including the primordia stage, ball-shaped stage, peach-shaped stage, and mature stage [3]. *P. impudicus* fruiting bodies are completely differentiated and edible in the peach-shaped stage, and can elongate to their full length and develop into mature fruiting bodies under suitable conditions in several hours [4]. Mature *P. impudicus* fruiting bodies are the main form consumed, but their quality deteriorates in a short period (above 4–6 h) due to aging and autolysis, so they should be harvested and processed within a few hours [3]. Peach-shaped *P. impudicus*, which is conducive to transport and storage, is harvested as an alternative for mature *P. impudicus*. Peach-shaped *P. impudicus* after harvest is usually stored and transported under low-temperature refrigeration, which consumes a large amount of energy, while some of the fruiting bodies cannot restart development after refrigerated storage, resulting in a waste of resources [5]. Therefore, effective methods to inhibit the peach-shaped *P. impudicus* maturation at room temperature are urgently needed.

Hexanal, a volatile C-6 aldehyde, has been approved by the Food and Drug Administration as a Generally Recognized as Safe food additive [6,7]. Hexanal treatment has been extensively studied in the field of postharvest preservation technology. It has been demonstrated that hexanal can extend the shelf life of various fruits and vegetables, for example, mango fruits [8], sweet bell peppers [9], pears [10], and fresh figs [11]. Phospholipase D (PLD), a ubiquitous phospholipid hydrolase in plant cells, can regulate various important cellular physiological functions and is a key enzyme in cell membrane damage during maturation and aging [12,13]. It has been shown that hexanal, an inhibitor of PLD, can delay the senescence of vegetables without damaging their color and flavors [9]. Some studies have explored the mechanism of hexanal treatment in extending the postharvest freshness of fruit and vegetables. For example, Kayal et al. [14] found that hexanal treatment prolonged the shelf life of strawberries and decreased the gene expression of PLD (PLD1 and PLD2). Moreover, hexanal treatment reduced the gene expression of PLD α, β, δ and ζ isoforms in peppers [15]. In addition to the PLD-inhibitory effect, hexanal also induced the downregulation of genes linked to ethylene signal transduction and cell wall degradation in tomatoes, indicating that hexanal may serve as a weak ethylene inhibitor [16]. In addition, Yumbya et al. [17] found that gene expression associated with ethylene biosynthesis and cell wall degradation was temporarily restricted after the hexanal treatment of bananas. Through transcriptome analysis, our previous study demonstrated that the phosphatidylinositol signaling system was important in the resumption of developmental processes in refrigerated peach-shaped *P. impudicus* and that the PLD may serve as a key gene in the regulation of maturation of peach-shaped *P. impudicus* [5]. Thus, it is speculated that hexanal may exert an efficacious regulatory influence on postharvest peach-shaped *P. impudicus* maturation.

This study aimed to evaluate mechanism by which hexanal treatments inhibited the peach-shaped *P. impudicus* maturation, and the efficacy of this process. Firstly, hexanal fumigation was used to treat peach-shaped *P. impudicus*, the effects of hexanal concentration and treatment time on the inhibition of peach-shaped *P. impudicus* maturation were investigated, and optimal hexanal treatment conditions were determined. Furthermore, the effects of hexanal treatment on gene expression and lipid abundance in peach-shaped *P. impudicus* were comprehensively explored using RNA sequencing and liquid chromatography–tandem mass spectrometry (LC-MS/MS) analysis to reveal the mechanism by which hexanal inhibited maturation. The research findings offer a theoretical foundation for developing hexanal treatment as a preservation technology for *P. impudicus*.

## 2. Materials and Methods

### 2.1. Materials

Peach-shaped *P. impudicus* was procured from a cultivated field in Kunming (Yunnan, China) and transported immediately to the laboratory at 4–10 °C following harvest. A TruseqTM RNA sample prep kit was obtained from Illumina Inc. (San Diego, CA, USA). TRIzol reagent was purchased from Thermo Fisher Scientific (Waltham, MA, USA). Hexanal, methanol, acetonitrile, isopropanol, and chloroform were obtained from Merck (Darmstadt, Germany). Methyl tert-butyl ether was purchased from CNW (Dusseldorf, Germany). Formic acid was obtained from Sigma-Aldrich (St. Louis, MO, USA). Ammonium formate was purchased from Fisher Scientific (Pittsburgh, PA, USA).

### 2.2. Optimization of Hexanal Concentration and Treatment Time

Each group comprised three biological replicates, which contained eight peach-shaped *P. impudicus*. The cleaned sample was immediately placed in a 13 L sealed plastic box, in which culture dishes containing different concentrations of hexanal (*V*_hexanal_: *V*_plastic box_ = 0, 25, 50, and 100 μL/L) and filter paper (to promote rapid evaporation) were placed, and then the plastic box was placed at room temperature for 8 h. Subsequently, hexanal-treated peach-shaped *P. impudicus* was placed under the specified conditions (26 ± 2 °C and 95 ± 3% relative humidity) for 3 days. After this period, the specimens were photographed, and the maturation rate was calculated. Peach-shaped *P. impudicus* treated with 0 μL/L hexanal served as a control group. In addition, to further analyze the effects of hexanal treatment time on the inhibition of peach-shaped *P. impudicus* maturation, the hexanal concentration was fixed at 25 μL/L. Specifically, the cleaned sample was immediately placed in a 13 L sealed plastic box, in which culture dishes containing 25 μL/L hexanal and filter paper were placed, and then the plastic box was placed at room temperature for varying treatment times (0, 4, 6, and 8 h). Subsequently, hexanal-treated peach-shaped *P. impudicus* was placed under the specified conditions (26 ± 2 °C and 95 ± 3% relative humidity) for 3 days. After this period, the specimens were photographed, and the maturation rate was calculated. Peach-shaped *P. impudicus* treated with 25 μL/L hexanal for 0 h served as a control group. Mature *P. impudicus* was characterized by stipe elongation and opening. The maturation rate was calculated as the ratio of the number of mature *P. impudicus* to the total number of *P. impudicus*, expressed as a percentage.

### 2.3. Transcriptome Analysis

#### 2.3.1. RNA Extraction and Detection

Six to eight samples were randomly selected from untreated peach-shaped *P. impudicus* (CK group), with mature *P. impudicus* (M-P group) obtained by placing peach-shaped *P. impudicus* under specified conditions for 3 days, and hexanal-treated peach-shaped *P. impudicus* (H-P-P group) obtained by placing peach-shaped *P. impudicus* under specified conditions for 3 days after 25 μL/L hexanal treatment for 6 h. Samples were washed and dried, and their stipes were collected and ground into a powder. The experiments on each group were conducted in triplicate. TRIzol reagent was utilized to extract the total RNA. The RNA concentration and purity were determined by a Nanodrop 2000 spectrophotometer (Thermo Scientific, Waltham, MA, USA). Agarose gel electrophoresis and Agilent5300 (Agilent Technologies, Santa Clara, CA, USA) were used for the detection of RNA integrity and RNA quality number, respectively.

#### 2.3.2. Transcriptome Sequencing and Assembly

The libraries were constructed by a TruseqTM RNA sample prep kit, and then total RNA was sequenced based on an Illumina NovaSeq 6000 sequencing platform [18]. The low-quality reads were filtered, and the clean data were acquired using the Fastp software (version 0.19.5) before being reassembled with the use of the Trinity software (version 2.8.5).

#### 2.3.3. Screening and Bioinformatics Analysis of DEG

The differentially expressed gene (DEG) was analyzed by the DESeq2 software (version 1.24.0), with a screening threshold set to |log2FC| ≥ 1 and *p*-adjust < 0.05. A pathway enrichment analysis was conducted utilizing the Kyoto Encyclopedia of Genes and Genomes (KEGG) database, with *p*-adjust < 0.05, which reflected significant enrichment in the KEGG pathway according to Fisher’s exact test.

### 2.4. Lipidomic Analysis

#### 2.4.1. Sample Preparation

Twenty milligrams of sample powder was accurately weighed from CK, M-P, and H-P-P groups each. Subsequently, 1 mL of a lipid extraction solution (*V*_methyl tert-butyl ether_: *V*_methanol_ = 3:1), which contained an internal standard, was added to the mixture and vortexed for 30 min. Ultrapure water (300 μL) was added to the mixture, vortexed for 1 min; then, it was placed in a 4 °C refrigerator for 10 min and centrifuged at 13,750× *g* for 3 min. Then, 400 μL supernatant was collected, and the solvent was evaporated at 20 °C. Finally, the samples were dissolved in 200 μL lipid-dissolving solutions (*V*_acetonitrile_: *V*_isopropanol_ = 1:1) and analyzed using LC-MS/MS. Experiments on each group were conducted in triplicate.

#### 2.4.2. LC-MS/MS Analysis

Samples were analyzed utilizing ExionLC™ AD ultra-performance liquid chromatography (UPLC) and QTRAP^®^ 6500+ tandem mass spectrometry (MS/MS). Chromatographic separation was conducted using a Thermo Accucore™ C30 column (2.6 μm, 2.1 mm × 100 mm i.d.). Mobile phase A comprised a 60% acetonitrile aqueous solution with 0.1% formic acid and 10 mmol/L ammonium formate, and mobile phase B consisted of an acetonitrile/isopropanol (10/90, *V/V*) solution, also containing formic acid and ammonium formate. Gradient elution was as follows: 80:20 A/B for 0 min, 70:30 A/B for 2 min, 40:60 A/B for 4 min, 15:85 A/B for 9 min, 10:90 A/B for 14 min, 5:95 A/B for 15.5 min, 5:95 A/B for 17.3 min, 80:20 A/B for 17.5 min and 80:20 A/B for 20 min. The injection volume, column temperature and flow rate were 2 μL, 45 °C and 0.35 mL/min, respectively.

MS was performed at an electrospray ionization temperature of 500 °C. The ion spray voltages were set to −4500 and 5500 kV. Ion source gas 1 (GS1) and GS2 were maintained at 45 and 55 psi, respectively. In triple-quadrupole MS mode, the ion pair was detected under the optimized declustering potential and collision energy.

#### 2.4.3. Quantitative Analysis of Lipids

A qualitative analysis of detected lipids was conducted by applying the Metware database (Metware Biotechnology Co., Ltd., Wuhan, China) according to the retention times and parent–daughter ion pair. A quantitative analysis of lipids was conducted by applying multiple reaction monitoring modes. Analyst software (version 1.6.3) was used to process the MS data, and the chromatographic peaks of substances in various samples were corrected. The actual content (abundance) of lipids (X, nmol/g) in the sample was calculated with following formula (1):

(1) X=0.001×R×c×F×V/m

where *R* represents the ratio of the sample peak area to that of the internal standard, *c* represents the internal standard concentration (μmol/L), *F* represents the internal standards correction factor of different substances, *m* represents the sample mass (g), and *V* represents the sample solution volume (μL).

### 2.5. Statistical Analysis

Data were presented as mean ± standard deviation. One-way analysis of variance (ANOVA) was conducted by GraphPad Prism software (version 8.0), with *p* < 0.05 considered statistically significant. Principal component analysis (PCA) was employed to elucidate lipid composition differences.

## 3. Results and Discussion

### 3.1. Effects of Hexanal Treatment on Maturation Rate of Peach-Shaped P. impudicus

**Figure 1:**
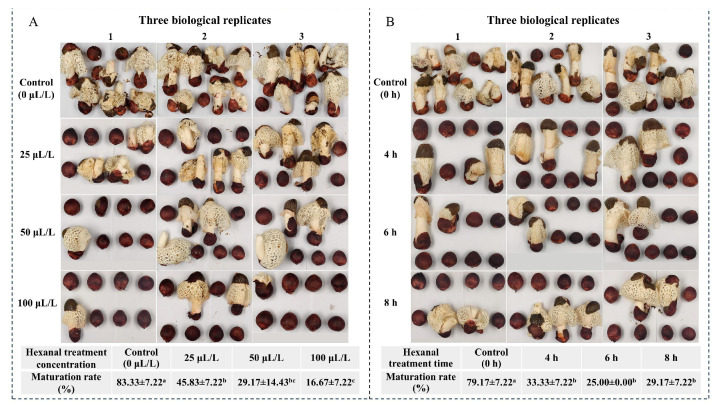
Maturation rate of peach-shaped *P. impudicus* after treatment with different concentrations of hexanal for 8 h (**A**) and treatment with 25 μL/L hexanal for different times (**B**). Different lowercase letters indicate significant differences between groups (*p* < 0.05).

The effect of hexanal treatment on inhibition of peach-shaped *P. impudicus* maturation was evaluated. Compared with the 0 μL/L hexanal treatment, treatment with different hexanal concentrations (25, 50, and 100 μL/L) for 8 h significantly inhibited peach-shaped *P. impudicus* maturation in a dose-dependent manner (*p* < 0.05) (Figure 1A). Furthermore, no significant differences were observed in the maturation rate of peach-shaped *P. impudicus* treated with 25 μL/L and 50 μL/L hexanal (*p* > 0.05). Therefore, the hexanal concentration was fixed at 25 μL/L to investigate the effects of varying treatment times on the maturation rate of peach-shaped *P. impudicus* (Figure 1B). Considering the difficulty of preservation, newly procured peach-shaped *P. impudicus* was used in each experimental batch. The hexanal treatment for different times (4, 6, and 8 h) significantly (*p* < 0.05) inhibited peach-shaped *P. impudicus* maturation, but no significant differences (*p* > 0.05) were observed among the different hexanal treatment times. Additionally, as shown in Figure 1A,B, the maturation rate of peach-shaped *P. impudicus* treatment with 25 μL/L hexanal for 8 h was 45.83% and 29.17%, respectively. This is due to the individual growth differences in *P. impudicus* in different batches. Notably, the inhibitory effect was most pronounced when the samples with treated with 25 μL/L hexanal for 6 h, resulting in a maturation rate of 25% for peach-shaped *P. impudicus*. Consequently, quantitative transcriptomic and lipidomic analyses were performed using peach-shaped *P. impudicus* treated with 25 μL/L hexanal for 6 h as experimental samples.

### 3.2. Transcriptome Analysis of Hexanal Inhibiting Peach-Shaped P. impudicus Maturation

#### 3.2.1. Sequencing Data and DEGs Analysis

**Figure 2:**
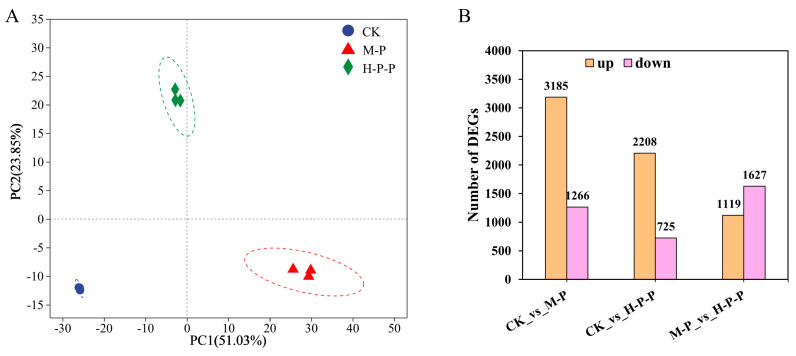
(**A**): Principal component analysis (PCA) among the CK, M-P, and H-P-P groups; (**B**): statistics diagram of differentially expressed genes (DEGs) in different comparison groups. CK: peach-shaped *P. impudicus*; M-P: mature *P. impudicus*; H-P-P: hexanal-treated peach-shaped *P. impudicus*.

The fruiting bodies of peach-shaped *P. impudicus*, treated with or without hexanal, exhibited immature peach-shaped *P. impudicus* and mature *P. impudicus* following storage under suitable conditions. Transcriptome sequencing was conducted on samples from the CK, M-P, and H-P-P groups to analyze the inhibitory effect of hexanal treatment on peach-shaped *P. impudicus* maturation. In total, 4,5351,198–58,574,626 clean reads were obtained. The error rate for each sample was less than 0.0271%, Q 30 was above 92.28%, and the GC percentage was between 46.92% and 47.48%. Furthermore, the PCA results demonstrated that the three biological replicates of each sample group were clustered on the distribution map (Figure 2A), indicating good data reproducibility. The CK group was separated from the M-P group in terms of the PC1 dimension and separated from the H-P-P group in terms of both the PC1 and PC2 dimensions, and the distance between the CK and H-P-P groups in terms of the PC1 dimension was smaller than that between the CK and M-P groups, indicating that hexanal treatment inhibited peach-shaped *P. impudicus* maturation at the transcriptional level.

In contrast to the CK group, 2208 DEGs were upregulated and 725 DEGs were downregulated in the H-P-P group (Figure 2B). In particular, 2746 DEGs were identified in the H-P-P group compared to the M-P group, and the number of downregulated DEGs (1627) was greater than that of upregulated DEGs (1119). The possible reason for this was that hexanal treatment inhibited the specific gene expression in peach-shaped *P. impudicus*, preventing maturation.

#### 3.2.2. KEGG Functional Enrichment Analysis of DEGs

Analysis of the signaling pathways could provide vital information elucidating the impact of hexanal on the developmental inhibition of peach-shaped *P. impudicus*. KEGG pathway enrichment results indicated that upregulated DEGs in the CK vs. H-P-P comparison were annotated to nine enriched pathways, including glycolysis/gluconeogenesis, the citrate cycle (TCA cycle), glutathione metabolism, methane metabolism, cysteine and methionine metabolism, clyoxylate and dicarboxylate metabolism, aflatoxin biosynthesis, tyrosine metabolism, and the pentose phosphate pathway. The downregulated DEGs in the M-P vs. H-P-P comparison were annotated to an enriched ribosome, while the upregulated DEGs were annotated to nine enriched pathways. Of these, except for the findings on glycolysis/gluconeogenesis and tyrosine metabolism, which were consistent with what was observed in the CK vs. H-P-P comparison, the others were involved in nicotinate and nicotinamide metabolism, thiamine metabolism, the MAPK signaling pathway—yeast, fatty acid degradation, atrazine degradation, β-Alanine metabolism, and tryptophan metabolism.

#### 3.2.3. Effects of Hexanal Treatment on Phosphatidylinositol Signaling System and MAPK Signaling Pathway

**Figure 3:**
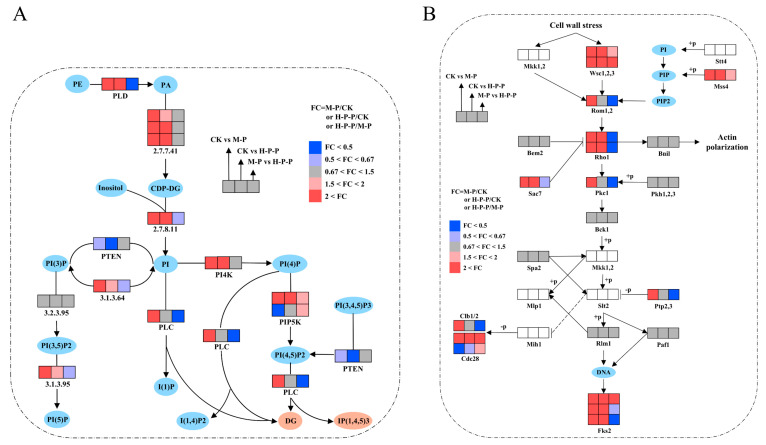
Schematic diagram of phosphatidylinositol signaling pathway (**A**) and cell wall integrity MAPK pathway (**B**).

Previous studies have demonstrated that the phosphatidylinositol signaling system and MAPK signaling pathway are crucial for the morphological development of peach-shaped *P. impudicus* [5]. The above pathways were enriched in the CK vs. H-P-P and M-P vs. H-P-P comparison, suggesting that hexanal treatment may inhibit the postharvest maturation of CK through these signaling pathways. Figure 3A illustrates that the gene expression of PLD was elevated 6.49- and 2.45-fold in M-P and H-P-P, respectively, in comparison to CK, while it was reduced 0.40-fold in H-P-P in contrast to M-P. Similarly, the gene expression of phosphatidylinositol phospholipase C (PLC), a key enzyme for producing signal molecules, was significantly upregulated in M-P compared with that in CK but was significantly downregulated 0.37-fold in H-P-P compared with M-P. These results suggested that hexanal treatment inhibited PLD gene expression, leading to a decrease in PLC gene expression, which in turn inhibited the activation of the phosphatidylinositol signaling system. Therefore, PLD may be involved in regulating CK maturation. As shown in Figure 3B, 14 DEGs were annotated in the cell wall integrity (CWI) MAPK pathway. In particular, Rom1,2, Clb2, Pkc1, Sac7, Ptp2,3, and Rho1 were significantly downregulated in the M-P vs. H-P-P comparison. Conversely, these key kinases or proteins were significantly upregulated in the CK vs. M-P comparison, suggesting that the CWI MAPK pathway was not activated in H-P-P, failing to amplify the signal cascade.

Hexanal, a potent inhibitor of PLD, improved the shelf life of fruits and vegetables by inhibiting PLD-mediated membrane catabolism. For example, Padmanabhan et al. [15] found that hexanal treatment inhibited the gene expression of PLD α, PLD β, PLD δ, and PLD ξ in pepper fruit, and the maturation of pepper fruits was delayed when they were exposed to hexanal for more than 6 h. Similarly, Arthika et al. [19] found that hexanal treatment significantly reduced the gene expression of PLD α, PLD ζ, and PLD γ in nectarines. We also demonstrated that hexanal treatment could significantly inhibit the PLD gene expression in peach-shaped *P. impudicus*. It has been demonstrated that when phosphatidic acid (PA, produced by PLD decomposing phospholipids) reaches a specific accumulation level, it can activate the phosphatidylinositol signaling system, resulting in a notable upregulation of the key enzyme PLC. Furthermore, the signal molecule diacylglycerol (DG), produced by PLC hydrolysis, can activate protein kinase C (PKC), in turn initiating the MAPK cascade reaction [20,21]. Thus, compared with that in M-P, PLD gene expression was inhibited in H-P-P, resulting in decreased PLC expression. As a result, the activation of the phosphatidylinositol signaling system and the signal amplification of MAPK-CWI were inhibited.

#### 3.2.4. Effects of Hexanal Treatment on Cell Wall Remodeling

Rapid stipe elongation is the most striking feature of the growth of CK, leading to its development into M-P [22]. During stipe elongation, the original glycosidic bonds in the cell wall are broken, and new cell wall components are introduced, thereby achieving cell wall expansion [23]. The gene expressions of enzymes associated with the primary components of the stipe cell wall (such as chitinase and β-1,3-glucosidase, etc.) were analyzed (Table 1) in this study. A comparison of CK with H-P-P revealed that two of the four chitinases were upregulated, while one of the six chitin synthases was downregulated. In total, 17 of the 24 β-glucanases (including β-glucosidase) were upregulated, and all 3 β-1,3-glucan synthases were upregulated. Interestingly, compared with those in M-P, approximately half of the β-glucanases, as well as chitinases and chitin synthases (except TRINITY_DN4936_c0_g1), in H-P-P were inhibited at the transcriptional level, suggesting that the maturation inhibition of peach-shaped *P. impudicus* by hexanal treatment may be closely related to β-glucanase and chitin-related enzymes, with the latter exerting a dominant influence. Kang et al. [24] demonstrated that the extension of the stipe cell wall resulted from the synergistic action of glucanase and chitinase. Therefore, these results indicated that the fruiting body failed to mature because the upstream signaling pathway regulating the morphological development of *P. impudicus* was not activated, resulting in downstream cell wall reconstruction-related enzymes failing to respond fully.

#### 3.2.5. Effects of Hexanal Treatment on Antioxidant Enzymes

Antioxidant enzymes, including superoxide dismutase (SOD), catalase (CAT), glutathione peroxidase (GPx), and peroxidase (POD), can maintain the redox balance in cells, prevent cell damage and effectively slow cell aging [25,26]. Further analysis was conducted to ascertain whether hexanal treatment affected the gene expression of antioxidant enzymes in peach-shaped *P. impudicus* (Table 2). Two CATs and nine SODs were annotated, and the gene expression of all antioxidant enzymes was upregulated in H-P-P compared to CK except TRINITY_DN989_c0_g1. In comparison with that in M-P, the gene expression of four SODs was downregulated in H-P-P, while the gene expression of two CATs and five SODs was upregulated. Tülin et al. [11] demonstrated that hexanal treatment increased CAT and POD activities in figs. Similarly, Cheema et al. [9] observed a significant increase in CAT and SOD activities in sweet bell pepper following hexanal treatment. Therefore, hexanal treatment can increase the gene expression of CAT and SOD, thereby maintaining a steady-state balance of reactive oxygen species and ultimately inhibiting the maturation of fruiting bodies under suitable conditions.

### 3.3. Lipidomic Analysis of Hexanal Inhibiting Peach-Shaped P. impudicus Maturation

#### 3.3.1. Overview of the Lipidome

Lipids, essential constituents of cells, perform critical functions in plant growth, morphogenesis, and the regulation of environmental stimuli [27]. A systematic analysis of the effects of hexanal treatment on the lipid species and abundance of peach-shaped *P. impudicus* helped elucidate the mechanism by which hexanal inhibited maturation. In this study, the sample quality control results showed the high stability of the mass spectrometer and the reliability of the data (Figure S1), which provided an essential guarantee for the repeatability and reliability of subsequent lipidomics analysis data.

**Figure 4:**
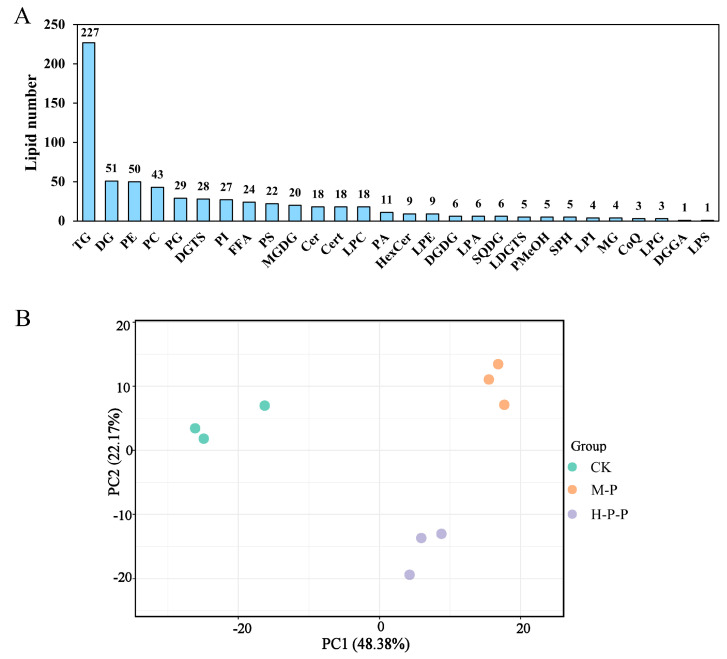
(**A**) Lipid numbers identified in the CK, M-P, and H-P-P groups; (**B**) principal component analysis (PCA). CK: peach-shaped *P. impudicus*; M-P: mature *P. impudicus*; H-P-P: hexanal-treated peach-shaped *P. impudicus*.

In total, 653 lipids were identified in the CK, M-P, and H-P-P groups, belonging to 28 lipid categories (Figure 4A), among which triglycerides (TG), diglycerides (DG), and phosphatidylethanolamines (PE) were more abundant, with 227, 51, and 50 species, respectively. Overall, differences in the lipidome profiles of the different samples were compared using PCA (Figure 4B). CK and M-P were significantly separated in terms of the PC1 dimension (48.38%), indicating that the maturation process profoundly impacted the lipidome of *P. impudicus*. Notably, H-P-P was significantly separated from CK in terms of the PC2 and PC1 dimensions and was significantly separated from M-P in the PC2 dimension, indicating that hexanal treatment induced alterations in the lipidome of peach-shaped *P. impudicus* in multiple dimensions.

#### 3.3.2. Abundance Changes of Lipid Categories and Differential Abundance of Lipids (DALs)

**Figure 5:**
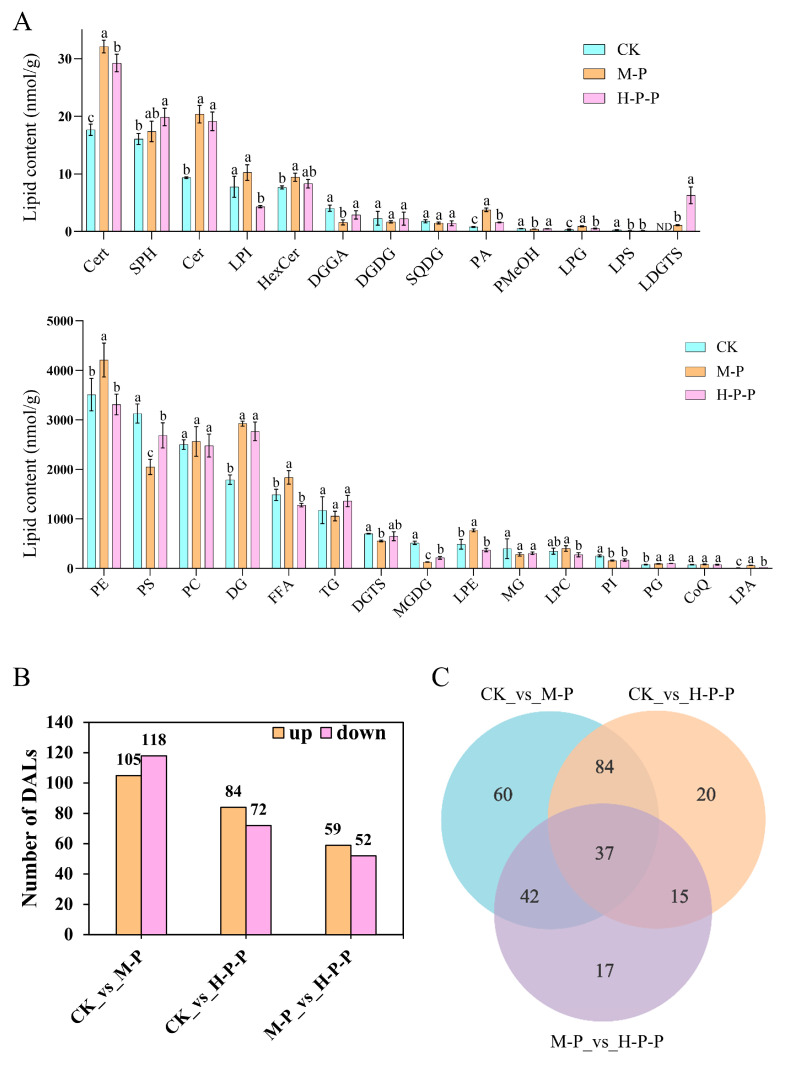
Changes in the abundance of lipid categories in the CK, M-P and H-P-P group (**A**); the statistics diagram (**B**) and the Venn diagram (**C**) for the differential abundance of lipids (DALs) in the pairwise comparison between groups. Different lowercase letters indicate significant differences between groups (*p* < 0.05).

The abundance changes of 28 lipid categories in the CK, M-P, and H-P-P groups were analyzed (Figure 5A). In comparison with those in CK, the abundance of PE, DG, free fatty acids (FFAs), lysophosphatidylethanolamines (LPEs), phosphatidylglycerols (PGs), lysophosphatidic acids (LPAs), phytoceramides (Certs), ceramides (Cers), hexosylceramides (HexCers), diacylglycerol glucuronic acid (DGGA), phosphatidic acids (PAs), and lysophosphatidylglycerols (LPGs) was significantly higher in M-P, and the abundance of phosphatidylserines (PSs), diacylgycerol-N-trimethylhomoserines (DGTSs), monogalactosyl diglycerides (MGDGs), phosphatidylinositols (PIs), diacylglycerol glucuronic acid (DGGA), phosphatidyl methanols (PMeOHs), and lysophosphatidylserine (LPS) was significantly lower (*p* < 0.05), indicating that the abundance changes of lipid categories in *P. impudicus* underwent notable changes during the ripening stage. The abundance of DG, PG, LPA, Cert, sphingosines (SPHs), Cer, PA, and LPG in H-P-P was significantly higher compared with that in CK (*p* < 0.05), while the abundance of PS, MGDG, PI, lysophosphatidylinositols (LPIs), and LPS was significantly lower. No statistically significant differences between M-P and H-P-P were observed in the abundance of DG, DGTS, PI, PG, SPH, Cer, HexCer, and LPS (*p* > 0.05). These results indicated the difference in abundance changes of lipid categories between hexanal-treated peach-shaped *P. impudicus* and mature *P. impudicus*, which aligned with the PCA findings (Figure 4B).

A total of 275 DALs were screened in pairwise comparisons of three *P. impudicus* lipidomes based on the variable importance in projection (VIP; VIP ≥ 1) combined with a fold change (FC ≥ 2.0 or FC ≤ 0.5). As shown in Figure 5B, 223 DALs were found in the CK compared with the M-P group. These DALs mainly included 93 TGs (18 up, 75 down), 13 MGDGs (13 down), 12 DGs (11 up and one down), and 12 PSs (7 up and five down), which may suggest *P. impudicus* undergoes potential lipid lipolysis and synthesis during maturation. The abundance of 156 (84 up and 72 down) lipids changed more significantly in the H-P-P group than in the CK group, and the majority were 55 TGs (21 up and 34 down) and 12 DGs (11 up and one down). In the M-P vs. H-P-P comparison, 111 (59 up, 52 down) DALs were screened, mainly including 34 TGs (33 up and one down), 9 DGTSs (9 up), and 9 LPCs (9 down). Apparently, there were significant changes in TG molecules in the CK vs. M-P, CK vs. H-P-P, and M-P vs. H-P-P comparisons, with more downregulated TGs than upregulated TGs in the first two comparisons. Conversely, the M-P vs. H-P-P comparison exhibited an inverse trend. This may indicate that hexanal treatment inhibited peach-shaped *P. impudicus* maturation at the lipidomic level. Additionally, 121 common and 35 unique DALs were identified between the CK vs. H-P-P and the CK vs. M-P comparisons (Figure 5C), potentially linked to the inhibition of peach-shaped *P. impudicus* maturation by hexanal.

#### 3.3.3. Effects of Hexanal Treatment on Phosphatidylinositol Signaling System at the Lipid Level

**Figure 6:**
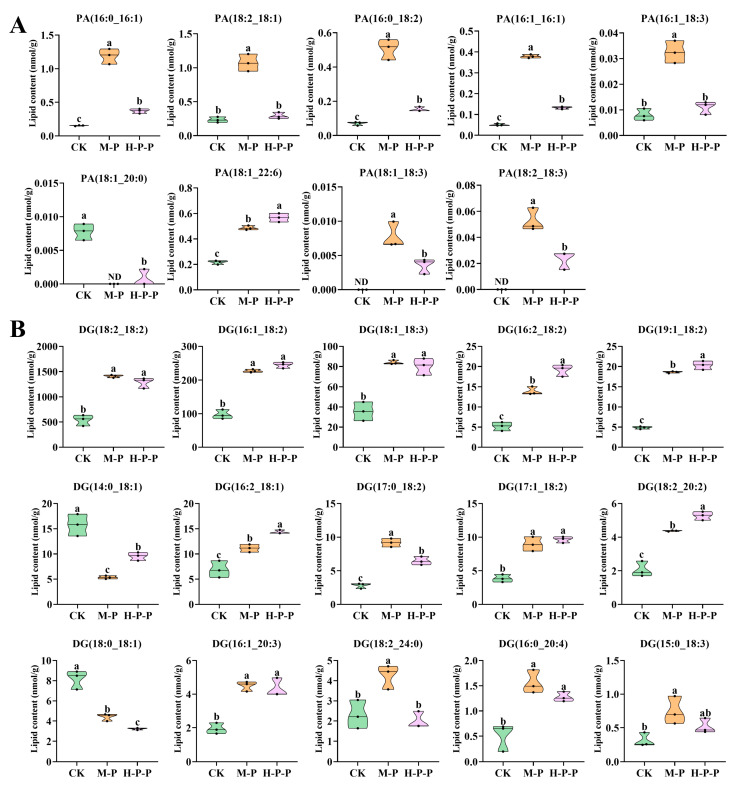
Changes in the abundance of representative differentially abundant lipid molecules in the phosphatidylinositol signaling pathway. (**A**) Phosphatidic acid (PA); (**B**) diacylglycerol (DG). Different lowercase letters indicate significant differences between groups (*p* < 0.05).

The KEGG enrichment results for DALs indicated that 19, 17, and 8 DALs were annotated to the CK vs. M-P, CK vs. H-P-P, and M-P vs. H-P-P comparison, respectively. These DALs belonged to the PA and DG categories. PA, the simplest glycerophospholipid, is a component of cell membranes, and its content varies with cell state [28]. It has been reported that a specific level of PA triggers PIP5K to generate PI(4,5)P2, which then activates the phosphatidylinositol signaling system [29]. As illustrated in Figure 6A, the abundance of PA(16:0_16:1), PA(18:2_18:1), PA(16:0_18:2), PA(16:1_16:1), PA(16:1_18:3), PA(18:1_18:3) and PA(18:2_18:3) in M-P was the highest, followed by H-P-P, and CK was the lowest. Moreover, no significant difference was observed in the abundance of PA(18:2_18:1) and PA(16:1_18:3) between H-P-P and CK (*p* > 0.05). This may be ascribed to the fact that hexanal treatment inhibited PLD gene expression (see Figure 3A), thereby reducing the abundance of PA molecules.

Diacylglycerol (DG) is a secondary messenger of the phosphatidylinositol (PI) signaling system, and can stimulate protein kinase C (PKC) to phosphorylate the substrate, thereby initiating the MAPK pathway [30,31]. The abundance of DG molecules in M-P was significantly higher than that in CK (*p* < 0.05), except for DG(14:0_18:1) and DG(18:0_18:1). However, except for DG(18:2_24:0), the abundance change trend of DG molecules in H-P-P and M-P was similar (Figure 6B). This result suggested that DG(18:2_24:0) is a pivotal signaling molecule that activates the MAPK pathway. Interestingly, the abundance of DG(16:2_18:2), DG(19:1_18:2), DG(14:0_18:1), DG(16:2_18:1) and DG(18:2_20:2) significantly increased and the abundance of DG(17:0_18:2), DG(18:0_18:1) and DG(18:2_24:0) significantly decreased (*p* < 0.05) in H-P-P compared with M-P, indicating that hexanal treatment may inhibit peach-shaped *P. impudicus* maturation through the above-mentioned DGs. In addition to being produced by the PLC hydrolysis of PI, DG can serve as an intermediate product for triglyceride synthesis [32]. The transcriptome results indicated that PLC gene expression was inhibited in H-P-P compared to M-P. This inhibition of the PLC gene could result in the accumulation of low levels of DG. However, no significant difference was observed in total DG abundance between H-P-P and M-P (*p* > 0.05), which may imply that DG was produced in a different way, i.e., via triglyceride synthesis.

#### 3.3.4. Effects of Hexanal Treatment on Cell Membranes of Stipes

During peach-shaped *P. impudicus* maturation, the cell wall of the stipe undergoes partial hydrolysis, which facilitates cell elongation [24]. Concurrently, the cell membrane displays dynamic changes in response to the cell elongation. Transcriptome results indicated that hexanal treatment inhibited cell wall reconstruction-related enzyme gene expression. Hexanal inhibited signal transduction by targeting PLD molecules on membrane lipids, impeding stipe elongation [9]. Therefore, a comprehensive analysis of the membrane lipids (glycerolipids, glycerophospholipids, and sphingolipids) of hexanal-treated peach-shaped *P. impudicus* was conducted.

**Figure 7:**
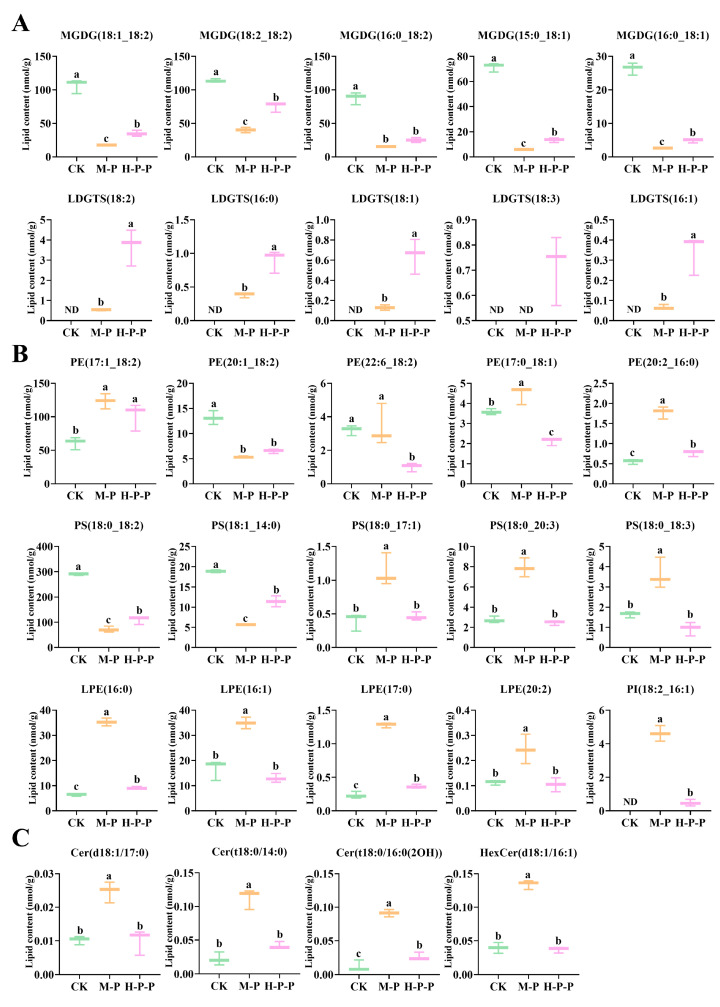
Changes in the abundance of representative differentially abundant lipid molecules in the cell membrane of the stipes of *P. impudicus*. (**A**) Glycerolipids; (**B**) glycerophospholipids; (**C**) sphingolipids. Different lowercase letters indicate significant differences between groups (*p* < 0.05).

(1) Glycerolipids. Nine glycerolipids (DG, TG, MG, DGTS, DGDG, DGGA, SQDG, MGDG, and LDGTS) were detected, among which the total abundance of DG, MGDG, and LDGTS was significantly different among different groups (*p* < 0.05) (Figure 5A). A discussion on DG was presented in Section 3.3.3, but in this section, the focus is on MGDG and LDGTS. MGDG is a non-bilayer lipid, accounting for approximately 50% of the total lipid composition in the thylakoid membranes of higher plants and green algae [33,34]. MGDG abundance was significantly higher in CK than in M-P or H-P-P, with M-P that in particular being the lowest (*p* < 0.05). The representative molecules of MGDG also showed a similar change trend (Figure 7A). Consequently, the maturation process resulted in a reduction in MGDG abundance in *P. impudicus*. Notably, hexanal treatment impeded this effect. In particular, the abundance of LDGTS representative molecules, (LDGTS(18:2), LDGTS(16:0), LDGTS(18:1), LDGTS(18:3), LDGTS(16:1)), in H-P-P was significantly higher than that in CK or M-P (*p* < 0.05). Previous studies have demonstrated that LDGTS isolated from microalgae can improve blood high-density lipoprotein functions [35]. Therefore, further investigation into the functional activity of LDGTS in *P. impudicus* is required. Studies have reported that reducing the MGDG/DGDG ratio could confer upon plants the capacity for stress adaptation by enhancing the ability to maintain membrane structure in a bilayer conformation [36]. Similarly, this study found that the MGDG/DGDG ratios of M-P (76.2) and H-P-P (95.9) were lower than those of CK (224.4), which suggested that peach-shaped *P. impudicus* treated with hexanal could adapt to changes in external pressure.
(2) Glycerophospholipids. In total, 13 glycerophospholipids (PE, PS, PC, PI, PG, PA, PMeOH, LPA, LPC, LPE, LPI, LPG, and LPS) were identified in CK, M-P and H-P-P. Among these, PE, PS, LPE, and PI exhibited the most pronounced differences between the various groups (Figure 5A). PEs, also known as phospholipids, are a crucial component of plant cell membranes. In comparison with those in M-P, the levels of PE(20:2_16:0), PE(22:6_18:2), and PE(17:0_18:1) were significantly lower in H-P-P (*p* < 0.05) (Figure 7B), indicating that three PE molecules may play a crucial role in cell membrane signaling, which explained why hexanal treatment lead to the downregulation of PLD gene expression in the stipes of *P. impudicus*. PS is usually located in the inner layer of the cell membrane and plays a role in various membrane-related functions [37]. The abundance of PS (18:0_18:3), PS (18:0_20:3) and PS (18:0_17:1) was significantly higher in M-P compared with that in CK (*p* < 0.05). The abundance of these PS molecules was significantly lower in H-P-P compared with that in M-P (*p* < 0.05), indicating that hexanal treatment significantly reduced the abundance of them, which played a pivotal role in stipe extension. PE reacted with serine to generate PS and ethanolamine under Ca^2+^ activation [38]. These results indicate that hexanal treatment might promote the process because H-P-P exhibited reduced PE abundance and increased PS abundance compared to M-P. LPE is a hydrolysis product of PE produced by phospholipase A [39]. The abundance of LPE(16:0), LPE(17:0), LPE(16:1), and LPE(20:2) in H-P-P was significantly lower than that in M-P (*p* < 0.05), which could be attributed to the hexanal treatment inhibiting PLA, which is similar to what was observed with PLD. PI plays a pivotal role in the phosphatidylinositol signaling system [40]. The abundance of PI (18:2_16:1) was significantly lower in H-P-P compared to that in M-P (*p* < 0.05). Thus, hexanal treatment inhibited *P. impudicus* maturation by inhibiting the phosphatidylinositol signaling system. Phospholipids are components of the lipid bilayer of membranes and are crucial in signal transduction [41].

Phospholipid signal transduction mainly occurs through phospholipases and lipid kinases that participate in specific phospholipid signaling pathways, thereby participating in the regulation of cells, including membrane deformation, membrane transport, and cytoskeleton rearrangement [42,43,44,45,46]. PC, PE, and PS are the main components of membrane phospholipids [47]. Previous studies have shown that the increasing PC/PE ratio can improve the stability of the membrane [48]. This study showed no significant differences in PC abundance among the CK, M-P, and H-P-P groups (*p* > 0.05). Notably, the PC/PE ratio of H-P-P (0.749) was greater than that of M-P (0.609), suggesting that hexanal treatment enhanced the stability of cell membranes.

(3) Sphingolipids. Sphingolipids are bioactive lipids of cell membranes, and numerous studies have shown that sphingolipids control key cellular functions, including the cell cycle, cellular senescence, apoptosis, migration, etc. [49,50]. Four sphingolipids (Cer, Cert, HexCer, and SPH) were identified (Figure 5A). Zhu et al. [51] demonstrated that Ca^2+^ in Pleurotus eryngii may affect the development of stipes via membrane-localized sphingolipid molecules. The total abundance of sphingolipids in M-P and H-P-P was significantly higher than that in CK (*p* < 0.05), indicating that sphingolipids played a role in signal transduction during *P. impudicus* maturation. Additionally, the abundance of Cer(d18:1/17:0), Cer(t18:0/14:0), Cer(t18:0/16:0(2OH)), and HexCer(d18:1/16:1) in M-P was significantly higher than that in CK (*p* < 0.05) (Figure 7C). In contrast, there was no significant difference between H-P-P and CK (*p* > 0.05), which indicated that the sphingolipids above were closely related to *P. impudicus* maturation, and hexanal treatment inhibited *P. impudicus* maturation by inhibiting the sphingolipids.

## 4. Conclusions

In this study, the inhibitory effects and mechanism of hexanal on peach-shaped *P. impudicus* maturation were analyzed. The results showed that the maturation rate of peach-shaped *P. impudicus* treated with 25 μL/L hexanal for 6 h was 25%. Transcriptome analysis results showed that the CK vs. H-P-P comparison had 2208 upregulated DEGs and 725 downregulated DEGs, and the M-P vs. H-P-P comparison had 1119 upregulated DEGs and 1627 downregulated DEGs. The hexanal treatment resulted in a reduction in PLD gene expression (the PLD gene expression in H-P-P was 0.40-fold lower than that in M-P), which had a negative effect on the signal transduction of the phosphatidylinositol signaling system and CWI MAPK pathway, thereby hindering the gene expression of β-glucanase and chitin-related enzymes. Lipidomic analysis showed that 653 lipid molecules were detected in the three groups. Of these, 275 were identified as DALs. The total abundance of PA and the abundance of representative PA molecules ((16:0_16:1), PA(16:1_16:1), PA(16:0_18:2), PA(16:1_18:3) and PA(18:2_18:1)) in hexanal-treated peach-shaped *P. impudicus* were lower than those in mature *P. impudicus*, suggesting that hexanal treatment reduced the accumulation of PA by inhibiting PLD gene expression, thereby inhibiting phosphatidylinositol signaling system activation. Moreover, hexanal treatment reduced the MGDG/DGDG ratio and increased the PC/PE ratio of peach-shaped *P. impudicus*, enhancing membrane stability. This study clarified that PLD was a key signaling molecule in the regulation of cell wall remodeling, providing key potential targets for the development of postharvest preservation technology for *P. impudicus*. Hexanal treatment may regulate the maturation of peach-shaped *P. impudicus* by inhibiting PLD gene expression and is easy to perform. Therefore, hexanal treatment is a promising postharvest preservation technology for peach-shaped *P. impudicus*.

## Figures and Tables

**Table 1:** Log2(fold change) of cell wall-related enzyme genes in the three comparison groups.

Type	Gene ID	Log2(Fold Change)		
		CK vs. M-P	CK vs. H-P-P	M-P vs. H-P-P
Chitinases	TRINITY_DN3514_c0_g1	0.61	−1.46	−1.98
	TRINITY_DN4688_c0_g1	0.90	−0.85	−1.65
	TRINITY_DN3122_c0_g1	2.40	1.16	−1.15
	TRINITY_DN4313_c0_g1	8.13	6.71	−1.32
Chitin synthases	TRINITY_DN4936_c0_g1	1.06	1.04	0.08
	TRINITY_DN31_c0_g1	1.15	0.36	−0.70
	TRINITY_DN244_c0_g1	1.33	0.26	−0.98
	TRINITY_DN1043_c0_g1	1.62	0.46	−1.07
	TRINITY_DN9021_c0_g1	−0.57	−1.86	−1.20
	TRINITY_DN4981_c0_g2	5.39	3.85	−1.45
β-1,3-Glucosidase	TRINITY_DN14093_c0_g1	3.59	7.93	4.49
	TRINITY_DN9019_c0_g1	5.43	6.64	1.32
	TRINITY_DN12787_c0_g1	6.59	7.80	1.31
	TRINITY_DN684_c0_g1	−1.53	−0.74	0.89
	TRINITY_DN1371_c0_g2	−1.94	−1.99	0.04
	TRINITY_DN4148_c1_g1	7.70	6.58	−1.03
	TRINITY_DN3011_c0_g1	1.28	0.03	−1.15
	TRINITY_DN10744_c0_g1	5.27	3.97	−1.20
	TRINITY_DN13997_c0_g1	8.22	3.09	−4.98
Endo-β-1,3(4)-glucanase	TRINITY_DN3286_c0_g1	−0.37	0.76	1.23
Endo-β-1,6-glucosidase	TRINITY_DN4364_c0_g2	8.02	4.20	−3.75
β-Glucosidase	TRINITY_DN8389_c1_g1	2.05	7.27	5.32
	TRINITY_DN738_c0_g1	−2.18	−1.32	0.95
	TRINITY_DN6295_c1_g2	8.27	8.38	0.21
	TRINITY_DN4234_c0_g3	6.90	6.54	−0.27
	TRINITY_DN576_c0_g1	1.06	0.18	−0.78
	TRINITY_DN12330_c0_g1	9.69	6.37	−3.21
Endo-β-1, 3 (4)-glucanase	TRINITY_DN1764_c0_g2	−1.88	−0.15	1.83
	TRINITY_DN858_c0_g1	−2.35	−1.06	1.39
	TRINITY_DN10744_c0_g1	5.27	3.97	−1.20
Endo-β-1,4-glucanase	TRINITY_DN4078_c0_g2	1.24	0.86	−0.29
	TRINITY_DN4535_c0_g1	−1.47	−1.86	−0.31
Endoglucanase-7	TRINITY_DN967_c0_g1	7.43	3.55	−3.78
Exoglucanase 3	TRINITY_DN2746_c0_g1	−1.24	−0.75	0.58
β-1,3-Glucan synthase	TRINITY_DN3479_c0_g3	1.67	0.85	−0.72
	TRINITY_DN5554_c0_g1	6.26	5.54	−0.63
	TRINITY_DN5554_c0_g2	6.01	4.32	−1.60

**Table 2:** Log2(fold change) of antioxidant enzyme genes in the three comparison groups.

Type	Gene ID	Log2(Fold Change)		
		CK vs. M-P	CK vs. H-P-P	M-P vs. H-P-P
Catalase	TRINITY_DN3676_c1_g1	−1.56	0.56	2.22
	TRINITY_DN9524_c0_g1	4.51	7.16	2.73
Superoxide dismutase	TRINITY_DN989_c0_g1	−2.12	−1.58	0.64
	TRINITY_DN2134_c0_g3	6.02	7.01	1.09
	TRINITY_DN7788_c0_g2	2.90	4.30	1.48
	TRINITY_DN2134_c0_g2	4.33	5.97	1.75
	TRINITY_DN3251_c0_g1	3.52	5.31	1.88
	TRINITY_DN10558_c0_g3	6.64	5.59	−0.95
	TRINITY_DN12621_c0_g1	7.00	3.77	−3.10
	TRINITY_DN11879_c0_g1	5.76	1.78	−3.85
	TRINITY_DN2134_c0_g4	5.87	1.77	−3.97

## Data Availability

The original contributions presented in the study are included in the article.

## Supplementary Materials

The following supporting information can be downloaded at: https://www.mdpi.com/article/10.3390/jof11020127/s1, Figure S1: Results of quality control (QC) sample analysis in the *P. impudicus* lipidomic analysis. Total ions current (TIC) plot for mass spectrometry analysis of the QC sample of the same mass in positive-ion mode (A) and negative-ion mode (B); Pearson correlation analysis of QC samples (C); Distribution of coefficient of variation (CV) values of QC samples (D). CK: peach-shaped *P. impudicus*; M-P: mature *P. impudicus*; H-P-P: hexanal-treated peach-shaped *P. impudicus*.
