# Supplementary material for: Inhibitory Effect and Mechanism of Hexanal on the Maturation of Peach-Shaped Phallus impudicus
Source: J Fungi (Basel). 2025 Feb 8;11(2):127. doi: 10.3390/jof11020127 (PMC11856996; doi:10.3390/jof11020127)
Supplement: Supplementary file 1 [file jof-11-00127-s001.zip › jof-3335047-supplementary.pdf]

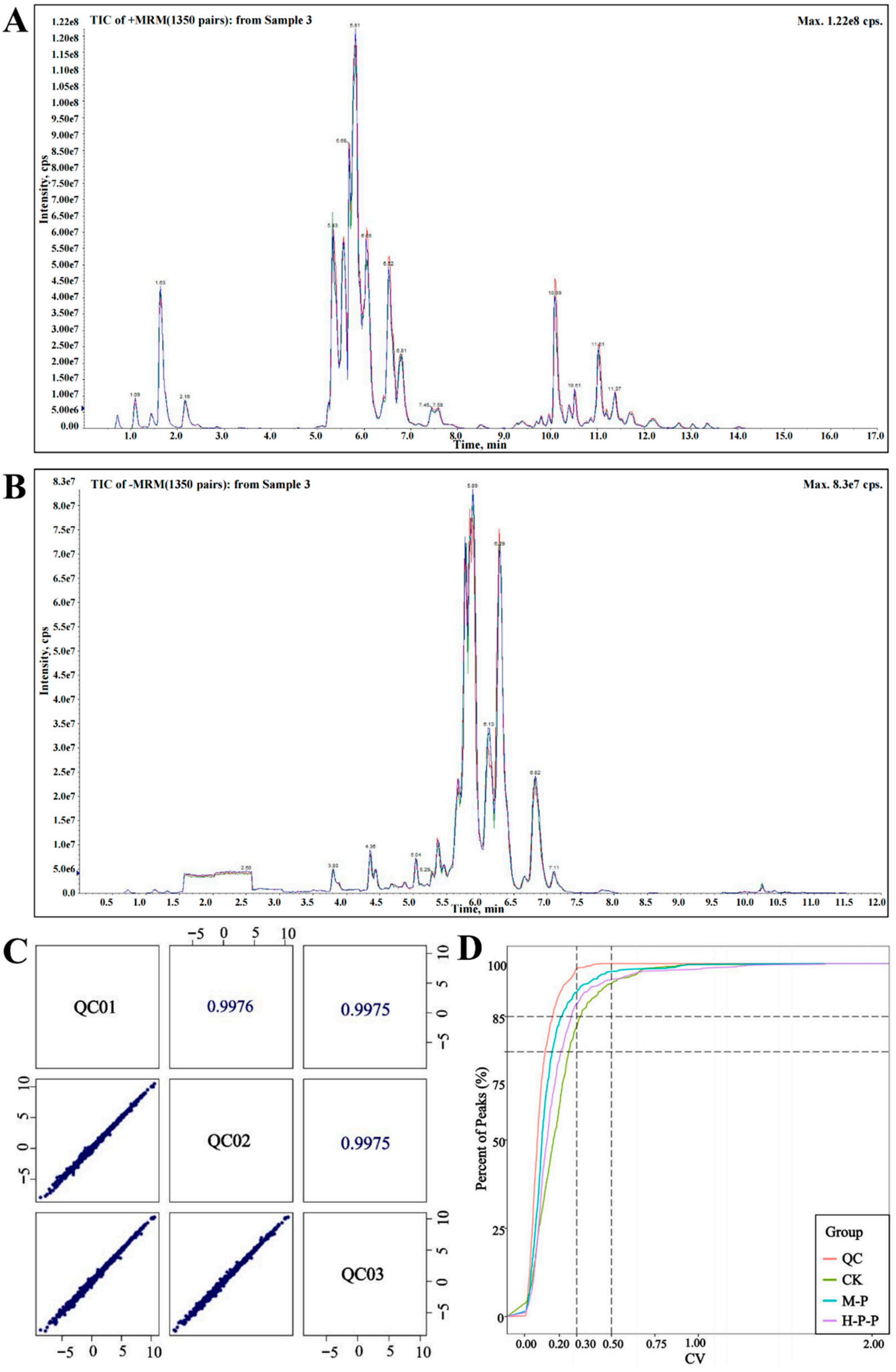

Figure S1. Results of quality control (QC) sample analysis in the *P. impudicus* lipidomic analysis. Total ions current (TIC) plot for mass spectrometry analysis of the QC sample of the same mass in positive-ion mode (A) and negative-ion mode (B); Pearson correlation analysis of QC samples (C); Distribution of coefficient of variation (CV) values of QC samples (D). CK: peach-shaped *P. impudicus*; M-P: mature *P. impudicus*; H-P-P: hexanal-treated peach-shaped *P. impudicus*.
